# Mechanism of human rhinovirus infections

**DOI:** 10.1186/s40348-016-0049-3

**Published:** 2016-06-01

**Authors:** Dieter Blaas, Renate Fuchs

**Affiliations:** Department of Pathophysiology and Allergy Research, Medical University of Vienna, Währinger Gürtel 18-20, 1090 Vienna, Austria; Department of Medical Biochemistry, Max F. Perutz Laboratories, Vienna Biocenter, Medical University of Vienna, Vienna, Austria

**Keywords:** Nasal epithelium, Human rhinoviruses, Rhinovirus receptors, Immune response

## Abstract

About 150 human rhinovirus serotypes are responsible for more than 50 % of recurrent upper respiratory infections. Despite having similar 3D structures, some bind members of the low-density lipoprotein receptor family, some ICAM-1, and some use CDHR3 for host cell infection. This is also reflected in the pathways exploited for cellular entry. We found that even rhinovirus serotypes binding the same receptor can travel along different endocytic pathways and release their RNA genome into the cytosol at different locations. How this may account for distinct immune responses elicited by various rhinoviruses and the observed symptoms of the common cold is briefly discussed.

## Introduction

Human rhinoviruses (HRVs) account for more than 50 % of upper respiratory tract infections. The disease is known as the common cold that usually resolves within 5–7 days. Symptoms include nasal stuffiness, sneezing, coughing, and a sore throat but about 12–32 % of HRV infections in children of less than 4 years are asymptomatic [1]. Treatment is so far only palliative as no vaccination and approved antivirals are available; because of the usually annoying but uncomplicated course of the disease, only drugs without side effects will be accepted by otherwise healthy patients. However, rhinovirus infections on top of chronic obstructive pulmonary disease (COPD), asthma, or cystic fibrosis (CF) might become life-threatening increasing the demand for the development of such antivirals [2].

Pre-school children can experience an upper respiratory infection up to 8 to 12 times per year (reviewed in [3]) that might lead to wheezing, otitis media, bronchiolitis, exacerbations of asthma, CF, or COPD and aggravate allergic reactions. The newly discovered RV-C species is thought to account for a significant proportion of HRV-related illness, especially in infants [4].

### The nasal epithelium

The main site of RV infections is the nasal mucosa. The nasal cavity is lined by a pseudostratified epithelium composed of columnar, ciliated epithelial cells, mucous-secreting goblet cells, and basal cells [5]; lymphocytes, mast cells, dendritic cells, and macrophages migrate to and then home in the epithelium under pathologic conditions. The epithelium is anchored in the underlying extracellular matrix that contains vascular endothelial cells and submucosal glands. The luminal, ciliary surface of the airways is covered by periciliary liquid and a mucus layer trapping inhaled particles such as bacteria and viruses. Mucus produced by the glands and goblet cells contains water, ions (e.g., Na^+^, Cl^−^, and K^+^), glycoproteins, and immunoglobulins such as IgG and polymeric IgA (pIgA) [6]. Beating cilia transport the mucus layer together with adhering particles to the oral cavity where it is swallowed; digestion then leads to destruction of the infectious agent. Mucociliary clearance requires a balance between ciliary beat, volume, and composition of mucus and periciliary fluid. This balance is perturbed in chronic inflammatory lung diseases such as CF and COPD. In CF, mucus composition, viscosity, and pH (a mean of 6.57 versus 7.18 in controls) are altered, rendering the airways more susceptible to infections [7].

### HRV receptors, entry, and replication

HRVs are non-enveloped with a ss(+)RNA genome that is protected by an icosahedral protein capsid built of 60 copies each of the four viral proteins VP1–VP4 [1]. Based on phylogeny, more than 150 HRV types are classified as species A, B, and C [8]. Twelve HRV-A (the minor group) bind members of the low-density lipoprotein receptor (LDLR) family whereas the remaining A and B types (the major group) bind intercellular adhesion molecule-1 (ICAM-1) [9, 10]; for HRV-C, the recently identified CDHR3 might serve as a receptor [11]. The mechanisms of entry and uncoating of HRV-C are unknown; we will thus limit the discussion to HRV-A and B.

For infection, the cognate receptor must be accessible to the virus, i.e., at the apical surface of ciliated epithelial cells. While reports on the location of ICAM-1 in the healthy nasal mucosa are contradictory, it is generally agreed that this receptor is upregulated upon inflammation [12]. Re-investigating this issue, we detected ICAM-1 at the ciliated surface of all nasal epithelial cells in the nasal tissue from healthy individuals (Ellinger et al., to be published). As expected from its “normal” physiologic function, LDLR is located at the basolateral plasma membrane of the polarized airway, intestinal, renal, and hepatic cell lines. We were thus surprised to find that LDLR and LDLR-related protein 1 (LRP-1) are present at the apical side of the nasal epithelial cells and thus available for uptake of virus at its main port of entry (Ellinger et al., to be published).

**Fig. 1:**
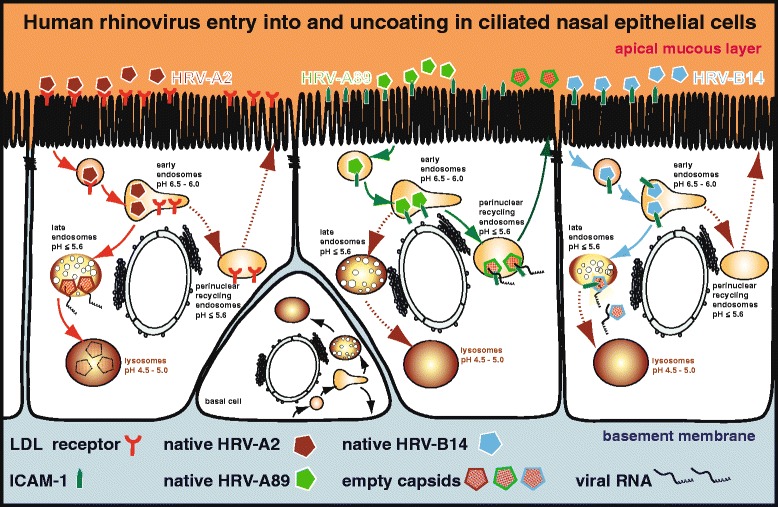
HRV entry and uncoating in ciliated nasal epithelial cells. *Left*, HRV-A2; *middle*, HRV-A89; and *right*, HRV-B14. For clarity, minor group and major group receptors (LDLR and ICAM-1, respectively) are depicted separately although they are co-expressed at the ciliated (apical) side of nasal epithelial cells as well as on the entire plasma membrane of basal cells. After binding to the respective receptor at the ciliated surface, the viruses are internalized and delivered into early endosomes. For HRV-A2, the mildly acidic pH in these compartments leads to dissociation of the virus from its receptor. While LDLR is returned to the apical plasma membrane via perinuclear recycling endosomes, the virus is directed to late endosomes. The low pH ≤ 5.6 in late endosomes converts native viruses into subviral A particles. Subsequently, the viral RNA is released and the remaining empty capsids (subviral B particles) are shuttled to and degraded in lysosomes. In contrast, HRV-A89 together with ICAM-1 is sorted into the recycling pathway. Perinuclear recycling endosomes are similarly acidic as late endosomes resulting in conversion of native HRV-A89 into A and then into B particles. After the transfer of the viral RNA into the cytoplasm, empty capsids are most likely returned to the apical mucous layer. After binding to the same receptor, HRV-B14 is routed from early endosomes into the pathway to lysosomes. However, after undergoing the ICAM-1-dependent conformational modification, the virus ruptures the endosomal membrane leading to RNA uncoating and its penetration into the cytoplasm. Thus, the viral capsid as well as the viral RNA escapes lysosomal degradation. So far, the fate of ICAM-1 is unknown

HRVs of species A and B investigated so far enter cells by receptor-mediated endocytosis [13]. In the endosomal lumen, they convert into subviral A (altered) particles devoid of the innermost capsid protein VP4 but still containing the RNA genome. After the release of the RNA (uncoating) into the cytoplasm, empty capsids remain (Fig. 1). Minor group HRVs exclusively depend on the low endosomal pH for this conformational modification and uncoating occurs even at 20 °C [13, 14]. Although uncoating of HRV-A2 is receptor-independent, the ß-propeller of LDLR and LRP plays a role in releasing the virus in early endosomes thus enabling its transport to late endosomes (pH ≤ 5.6), a station most suitable for RNA transfer into the cytosol [13]. On the other hand, it is generally accepted that conversion of ICAM-1 binding HRVs into A particles is facilitated by the receptor above 26 °C [15]; in addition, depending on the serotype, the process also requires low endosomal pH [16]. However, major group HRV-A89 can also convert at 20 °C in a low pH-dependent but presumably receptor-independent manner and, even more importantly, it follows a route different from the one taken by HRV-B14 (Conzemius et al., manuscript submitted). While HRV-A89 productively uncoats in the perinuclear recycling compartment, HRV-B14 penetrates into the cytoplasm by rupturing endosomes en route to the lysosomes in a temperature- (≥20 °C), low-pH, and ICAM-1-dependent manner (Fig. 1). Whether this is related to different affinity of ICAM-1 for the respective virus—and associated differences in dissociation of the virus-receptor complex—is currently under investigation.

Once the RNA has arrived in the cytoplasm, it is translated into a polyprotein. After autocatalytic cleavage into the structural (capsid) and non-structural proteins, the RNA is replicated by the viral polymerase. Finally, infectious progeny is assembled and released into the nasal cavity [1]. In contrast to HRV infection in tissue culture cells, airway epithelial cells of patients are not lysed for virus release; as shown for other enteroviruses, it is thus possible that cell-to-cell spread might occur via virus-carrying microvesicles [17].

### Host response to HRV infections

Although initially believed that HRV infection was limited to the upper airways, replicating virus was found in ciliated epithelial cells of the lower respiratory tract. Infected cells appear in patches, and only 10 % of the ciliated cells produce viral proteins and RNA [18]. Similar results had been obtained with in situ hybridization in nasal biopsies, again indicating that only a small proportion of cells were infected [19]. Nevertheless, basal cells are more susceptible to infection as compared to fully differentiated ciliated cells [20]. This might be related to the higher expression level of ICAM-1 in basal cells versus ciliated cells (Fuchs, unpublished observations). The absence of visible cytopathic alterations in the airway epithelium led to the hypothesis that the symptoms are rather due to the immune response of the host [21]. Upon HRV entry into and replication in ciliated epithelial cells, signalling pathways are activated leading to the release of various cytokines (IL-1ß, TNF, IL-8, IL-6, IL-11), chemokines (Rantes, MCP-1, MP-10), vasoactive peptides (bradykinin), and growth factors (VFGF) [21]. Consequently, inflammatory cells (leukocytes, granulocytes, monocytes) become activated and invade the submucosa. This results in amplification of the inflammatory process and the typical symptoms of the common cold. Conversely, HRV infections are controlled by innate and adaptive immune responses. Type-I interferons are the early mediators of the innate immune system, while neutralizing IgA and IgG in serum and secretions are observed 1–2 weeks after infection as a consequence of the adaptive immune response. Nasal epithelial cells express the pIgA receptor (pIgR) and the neonatal Fc-receptor (FcRn) that transport the respective immunoglobulins into nasal secretions [22]. Expression of FcRn in the ciliated epithelial cells and in dendritic cells (DC) in the nasal mucosa might contribute to mucosal immunity as shown for FcRn in the intestine [23].

How could the entry pathway taken by a given rhinovirus impact on the immune response? We presented evidence for HRV-A2 transferring its genome into the cytosol via a pore in the membrane and the remaining empty capsid being directed towards lysosomes where it is degraded. On the other hand, HRV-B14 breaks the endosomal membrane resulting in arrival of viral proteins in the cytoplasm [13]. As a consequence, one might hypothesize that the proteins of incoming virus are presented to the immune system either as products of proteasomal (HRV-B14) or lysosomal (HRV-A2) processing. Degradation products of the former would thus be mainly presented by the MHC-I system and the latter mainly by the MHC-II system. Furthermore, pattern recognition receptors in the endosome are different from those in the cytosol [1]. Such differences might impact on differences in the primary immune response; nevertheless, in the absence of further experimentation, this remains pure speculation.

### Summary and conclusions

HRVs are a major cause of respiratory infections of infants. The numerous serotypes are precluding the development of a vaccine, and current treatments only palliate the symptoms. HRVs enter from the apical side of the cells lining the airways by receptor-mediated endocytosis via LDLR and ICAM-1. We demonstrated that all nasal epithelial cells express ICAM-1 and LDLR at their ciliated side and that two ICAM-1 binding HRV types exhibit different temperature dependence of uncoating and take distinct routes inside the cell for genome release (Fig. 1). This may explain why different HRVs elicit different signals during cell entry resulting in different host responses to infection [24, 25]. Understanding the details of receptor binding, entry, and uncoating is crucial for identifying novel means of fighting the common cold.
